# A Silicon UCN Detector With Large Area and With Analysis of UCN Polarization

**DOI:** 10.6028/jres.110.041

**Published:** 2005-06-01

**Authors:** M. Lasakov, A. Serebrov, A. Khusainov, A. Pustovoit, Yu. Borisov, A. Fomin, P. Geltenbort, O. Kon’kov, I. Kotina, A. Shablii, V. Solovei, A. Vasiliev

**Affiliations:** St. Petersburg Nuclear Physics Institute, Gatchina, Russia; Institut Max von Laue – Paul Langevin, Grenoble, France; Ioffe Physical Technical Institute, St. Petersburg, Russia; St. Petersburg Nuclear Physics Institute, Gatchina, Russia

**Keywords:** ultracold neutrons

## Abstract

A silicon ultracold neutron (UCN) detector with an area of 45 cm^2^ and with a ^6^LiF converter is developed at St. Petersburg Nuclear Physics Institute (PNPI). The spectral efficiency of the silicon UCN detector was measured by means of a gravitational spectrometer at Institut Max von Laue – Paul Langevin (ILL). The sandwich-type detector from two silicon plates with a ^6^LiF converter placed between them was also studied. Using this type of technology the UCN detector with analysis of polarization was developed and tested. The analyzing power of this detector assembly reaches up 75 % for the main part of UCN spectrum. This UCN detector with analysis of UCN polarization can be used in the new electric dipole moment (EDM) spectrometer.

## 1. A Si UCN Detector With Large Area and Measurement of Its Energy Dependent Efficiency

For the production of the Si UCN detector with large area we used a Si wafer of 78 mm diameter. The area of the detector (48 cm^2^) was divided into four parts to decrease the capacity of the detector. It allows one to keep the amplitude of the signal at a sufficiently high level with respect to detector noise. The surface of the detector was coated with ^6^LiF (80 % ^6^Li enrichment) with a thickness of 0.6 mg/cm^2^ to 0.8 mg/cm^2^. The energy dependence of the registration efficiency was measured using the gravitational spectrometer.

**Fig. 1:**
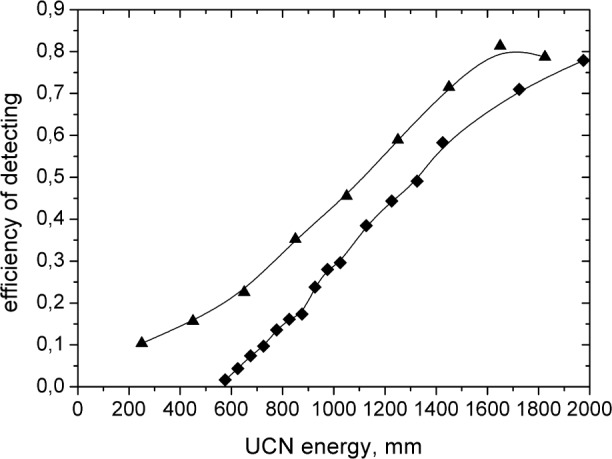
Energy dependence of registration efficiency: ■^3^He UCN detector with Al window (100 μm); ▲Si detector with ^6^LiF (80 % ^6^Li enrichment) converter on its surface.

Figure 1 presents the energy dependence of the registration efficiency for the ^3^He UCN detector with an Al window (100 µm thickness) and for the Si detector with a single ^6^LiF (80 % ^6^Li enrichment) converter on its surface. Efficiencies of both detectors were compared in the upper part of the spectrum when detectors were in the down position.

The efficiency of the Si detector is better than that of the ^3^He detector although the boundary velocity of ^6^LiF (80 % ^6^Li enrichment) is higher than the boundary velocity of the ^3^He detector Al window. It is connected with that the absorption coefficient of materials with a high capture cross section is sufficiently bigger for the low energy region and that the density of coated ^6^LiF is 30 % lower than the density of a LiF crystal [1].

## 2. A Si Sandwich UCN Detector Two Si Wafers and a ^6^With LiF Converter

This detector was developed at PNPI and investigated in an experiment at ILL. It allowed one to obtain the fraction of α-particles lost in the ^6^LiF converter by signal summation from the first and the second detectors and application of coincidence and anticoincidence techniques.

The fraction of signals without coincidence is about 28 % and corresponds to events for which the triton was registered and the α-particle was absorbed in the ^6^LiF converter. The thickness of the converter was between 0.6 mg/cm^2^ and 0.8 mg/cm^2^.

The results of the measurements allow us to conclude that the detector with one Si wafer and ^6^LiF converter looses 14 % of events due to α-particle absorption in the ^6^LiF converter. Thus, the registration efficiency of this detector is about 86 %. This conclusion does not contradict with the results of the registration efficiency measurements carried out with the gravitational spectrometer.

The Si sandwich UCN detector is fully efficient for tritons, but decreases the flux of falling UCN with energies of 200 neV to 300 neV by about 20 % to 25 %. Thus, the Si sandwich detector has 75 % to 80 % efficiency for UCN with energies of 200 neV to 300 neV. It should be used with a vertical guide to accelerate UCN in the gravitational field, like the ^3^He detector with an Al window.

## 3. A Si UCN Detector With Polarization Analysis

**Fig. 2:**
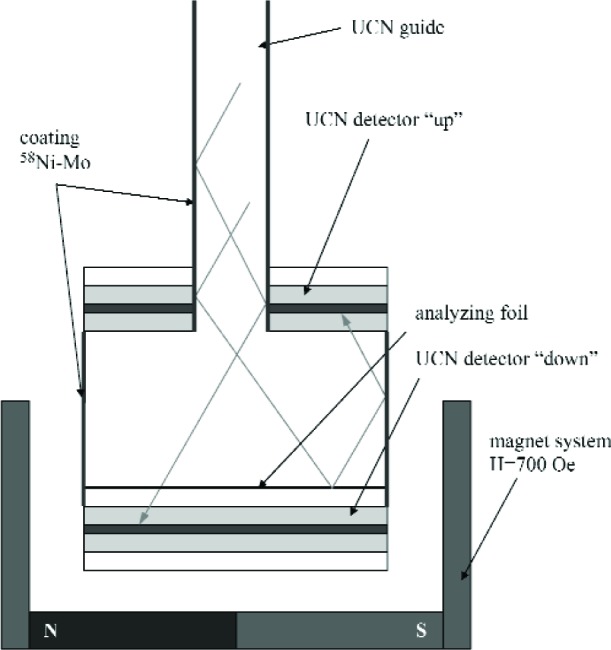
Scheme of Si UCN detector with polarization analysis.

The scheme of this detector is presented in Fig. 2. The detector consists of an upper detector for registration of the “up” spin component and a down detector for registration of the “down” spin component. “Up” and “down” polarization components are defined in compliance with the position of the neutron energy levels in the magnetic field. A magnetized ferromagnetic film is placed between the detectors which reflects the “up” spin component and allows the “down” spin component to pass. The down detector is a mosaic of five single detectors 2 cm × 6 cm. The upper detector consists of four single detectors 2 cm × 6 cm grouped around the neutron guide. The area of the upper detector is 20 % less than the area of the down detector and a part of the reflected neutrons returns to the neutron guide. These neutrons have a chance to return and to be analyzed again.

The analyzing power of the detector can be defined by the polarizing ratio:

$$R=\frac{N_{d}-N_{\text{up}}}{N_{d}+N_{\text{up}}}=P\cdot A,$$ (1)

where *N*_up_ is the count rate of the upper detector, *N*_d_ is the count rate of the down detector, *P* is the UCN polarization, and *A* is the analyzing power. In the ideal case this ratio is equal to 1 when the flux is completely polarized (the superconducting solenoid-polarizer is switched on) and it is equal to 0 when the flux is unpolarized (the solenoid is switched off). In reality there are several circumstances which disturb these correlations: the registration efficiencies of the detectors are different because of different areas; there is an effect of reflection of UCN from materials which does not depend on the polarization of the neutrons and decreases the efficiency of the polarization analysis; and the analyzing power of the detector depends on the properties of the analyzing ferromagnetic foil. Different materials which could be used as a substrate for thin ferromagnetic films were studied: Al foils (annealed and unannealed) and Si wafers.

**Fig. 3:**
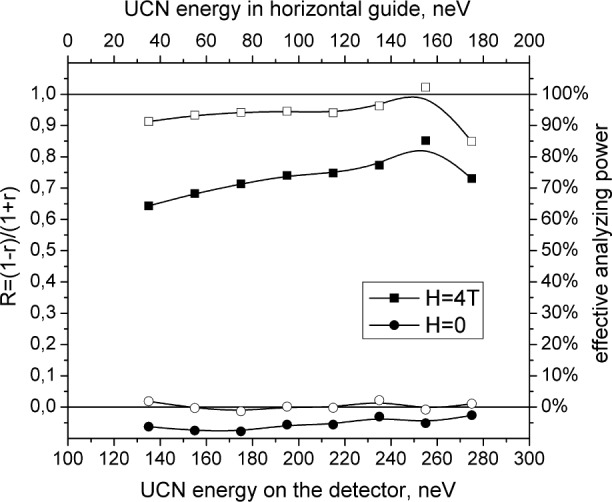
Energy dependence of polarizing ratio: ■ are for flux with full polarization (solenoid is switched on, *H* = 4 T); ● are for unpolarized flux (solenoid is switched off, *H* = 0 T). □ and ○ correspond to the values corrected for the albedo effect.

Figure 3 shows energy dependence of the polarizing ratio (1) for the flux with the full polarization (solenoid is switched on, *H* = 4 T) and for unpolarized flux (solenoid is switched off, *H* = 0 T). When we change the UCN polarization from 1 to 0, the polarizing ratio is changed by 0.8. Overall this result is not bad. The imperfection in splitting of spin components is connected with the effect of UCN reflection from the detector and the substrate of the analyzer. Measurements of the detector count rate ratios with an Al substrate but without ferromagnetic film showed that the albedo effect is determinative. Using the ratio obtained in these measurements as a correction allows one to reconstruct almost completely the full polarization value. The empty squares and circles are polarization ratios for polarized and unpolarized flux after correcting for the albedo effect.

In conclusion, the newly developed UCN detector with polarization analysis is very promising, e.g., for an application in the nEDM experiment. Obtained parameters of this detector are sufficient to solve the task, but further improvements are expedient.
